# Contextualizing toxic elements in the diet: a case for integration of toxic element data into food databases

**DOI:** 10.3389/fnut.2024.1473282

**Published:** 2024-09-18

**Authors:** Rachel R. Jones, Melanie T. Odenkirk, Jackie Bertoldo, Jessica E. Prenni

**Affiliations:** ^1^Department of Horticulture and Landscape Architecture, Colorado State University, Fort Collins, CO, United States; ^2^American Heart Association, New York, NY, United States

**Keywords:** toxic elements, food composition databases, food composition analysis, nutrition practice, food safety

## Abstract

Food composition data plays a key role in the practice of nutrition. However, nutrition professionals may currently lack the resources they need to integrate information about toxic elements – such as arsenic, cadmium, and lead – in food into the advice they give consumers. Geographic, sociocultural, and individual factors may impact not only the toxic element content of food, but also how the balance between potentially toxic and health-promoting components of food must be weighed. Better integration and contextualization of toxic element data into key food databases could allow for more nuanced, comprehensive nutrition guidance.

## 1 Introduction

Food comprises a vast array of diverse chemicals, from carbohydrates to vitamins to fatty acids. Of these constituents, the structurally simplest are the chemical elements. However, the 118 elements currently in the periodic table still differ from one another along many axes. Elements are broadly grouped into (several types of) metals, metalloids, lanthanides, actinides, halogens, noble gasses, and other nonmetals. Across these groupings, the concentrations of elements in foods can vary over at least 6 orders of magnitude, with macrominerals like sodium and potassium generally present at relatively high levels (10s of mg per g of food on a dry weight basis) and ultratrace elements like boron and nickel generally present at relatively low levels (10s of μg per g of food) (1). Considerations around dietary intake of elements also vary. While some elements, like zinc and iron, are essential for good nutrition, others, like arsenic and lead, can be toxic.

Consumers in the United States are used to seeing four nutritive elements (sodium, calcium, iron, and potassium) on nutrition facts labels. These labels do not – and cannot – communicate the vast chemical diversity contained in food. Nutritional databases, such as the United States Department of Agriculture’s FoodData Central (2), offer an expanded view of nutritionally important elements in commonly consumed foods by also including magnesium, phosphorus, zinc, copper, and manganese (among others). These data are frequently used to estimate average intakes of these nutrients across populations to evaluate health impacts and generate nutritional guidance. However, we argue that the absence of comprehensive, robust, and easily accessible information about toxic metals and metalloids (such as arsenic, cadmium, and lead) in food creates a significant shortfall in nutrition education both for professionals and for members of the public. While high acute exposure to toxic elements can cause poisoning and even death (3–5), for the average consumer in the United States, food typically does not pose such acute concerns. (Significant contamination events can occur; for example, the US Food and Drug Administration recently investigated cinnamon applesauce products high in lead and chromium (6)). However, the health risks of lower-dose chronic exposure to these metals from the diet is an important public health consideration. Inorganic arsenic (i.e., chemical forms in which the metalloid is not bound to carbon) is a known carcinogen (4, 7), cadmium can damage kidney function (3), and the impacts of chronic lead exposure can be both wide-ranging and particularly deleterious for children and pregnant individuals (5). Importantly, depending on dietary intake, some metals can be passed from the body of a lactating individual to an infant (8). It also seems likely that a variety of heavy metals can contribute to the etiology of cardiovascular disease (9).

Beyond these established effects, research suggests potential links between numerous other diseases states and toxic metals. Although the literature has not provided definitive conclusions on suggested connections between metabolic syndrome and intake of toxic metals (10), correlations (both positive and negative) have been identified in several retrospective analyses of the United States (11, 12) and Korean (13) National Health Examination Surveys. These studies have reported that the relationship between toxic metal exposure and metabolic syndrome may be nonlinear (12) and vary by metal (11), and that exposure to multiple metals may be more deleterious than the sum of risks for individual metals would suggest (13) (see also Liu et al.’s more recent work on this topic (14)). Negative impacts on immune function have also been linked to several toxic elements (15) (for an in-depth review of cadmium’s effects, see Wang et al. (16)).

**Figure 1:**
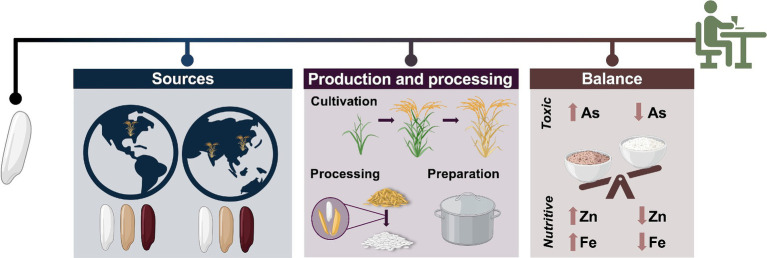
An overview of factors that may influence dietary considerations related to toxic element composition of food, using rice as an example. Different rice sources (geographic and genetic) may accumulate different levels of toxic elements. Cultivation practices, processing techniques, and preparation methods can also impact toxic element composition. The benefits of approaches that can reduce dietary toxic element intake, such as polishing brown rice to make white rice, must be weighed against their potential nutritional costs. (Created in BioRender. Prenni, J. (2024) BioRender.com).

Toxic metals in food come from the environments (soil, water, and air) where foods are produced. Toxic metals occur naturally throughout these environments and may also be introduced through industrial activity (17). Concentrations of toxic metals vary substantially across landscapes and methods of food production, and crops differ from one another in their tendencies to accumulate these metals (17). This variability can make it difficult to estimate population levels of toxic metal exposure through food consumption and to generate meaningful consumer guidance on how to minimize toxic metal exposure in their diets. Independent analyses by the United States FDA (6) and the nonprofit organization Consumer Reports (18) have elevated concerns of toxic metal contamination in various foods into public discourse. However, interpreting the implications of these types of studies and directing future research in this space requires a nuanced approach. The ubiquity and variability of toxic metals across the food supply necessitates an evaluation of trade-offs between the health-promoting effects of certain foods and the risk associated with toxic metal exposure at the dietary level. Important questions that need to be answered more comprehensively include (Figure 1): *What sources of commonly consumed foods are associated with meaningful levels of toxic metals? How might different sourcing or production strategies mitigate the prevalence of toxic metals in the food supply? What are the potential health benefits and tradeoffs of consuming different types and amounts of foods, given the balance of beneficial and potentially harmful compounds contained therein?*

## 2 Availability and utility of data on toxic metals in food

For food composition data to inform nutritional guidance, the data must be available and accessible to professionals who provide consumer dietary advice, such as physicians and dietitians. To the best of our understanding, while training in the medical and dietetics fields may include some discussion of dietary toxic metals, there is a lack of emphasis on the integration of this knowledge into clinical practice. Further exacerbating the problem, of the primary food composition databases available to US nutrition professionals (19), only the FDA Total Diet Study (TDS) includes acutely toxic elements such as arsenic and lead. (FoodData Central does incorporate a number of elements like selenium and copper that, though nutritionally relevant at low levels, may be concerning at high levels (2)). Although the TDS is a wealth of valuable information and the FDA provides a helpful summary report (20), if the user wishes to interact with all the food-level data it appears they must do so through a flat file that has approximately 69,000 rows (21). Unless the user has coding or Excel programming experience, extracting information from these files would likely be challenging. Internationally, some large food databases do integrate information about acutely toxic elements. The Canadian FooDB project incorporates arsenic, cadmium, and lead alongside a wide range of nutritive food components (22). It is important to consider the sources of this data to evaluate its relevance across contexts. A few foods are linked with specific references to published literature; many foods seem to be associated with other databases. One key citation for quantitative arsenic, cadmium, and lead information is to a USDA plant database that, while rich, only offers insight into plant foods and seems to largely reference toxic element data that is now 25 or more years old (23). To the best of our understanding, another key reference seems to be Frida, the Danish Food Composition Database, which does incorporate arsenic, cadmium, and lead values in a variety of foods and was last updated in May 2024 (24). The Australian Food Composition Database also includes arsenic, cadmium and lead (25). The aforementioned influence of environmental factors on foods’ toxic element content suggests that these example resources are unlikely to offer complete solutions for all nutrition professionals. However, such resources do demonstrate the feasibility of further expanding databases already familiar to many United States providers.

Although the US FDA does test for toxic metals in the food supply and has a Toxic Elements Working Group (26), currently another data challenge stems from an apparent lack of clear tolerable dietary intake levels within United States policy for several acutely toxic metals. The Food and Agriculture Organization (FAO) /World Health Organization (WHO) Joint Expert Committee on Food Additives publishes a searchable database of food additives and contaminants, which provides provisional tolerable intakes for cadmium and (methyl) mercury, but such values are not currently available for inorganic arsenic and seem unlikely to be feasible for lead (27). There are also some sources of food-level guidance around certain toxic metals. Among the toxic metals measured in the FDA TDS, only lead in apple juice and candy and inorganic arsenic in apple juice and infant rice cereal are assigned action levels (20). The FAO and WHO publish the Codex Alimentarius, which contains standards suggesting maximum allowable levels of (inorganic) arsenic, cadmium, lead, (methyl) mercury, and tin in a variety of foods that may be part of international trade (28). Nutrition professionals should be aware of these important resources, but it is not clear how a provider should triangulate across them and integrate individual and social factors to determine the best advice for many patients.

Further complicating this calculation are cases in which the risk of harm from toxic metals must be balanced with the benefits provided by otherwise nutritious foods. For example, while spinach is widely understood to be a healthy food, this crop may also be particularly prone to accumulate cadmium (1, 29). Similarly, in wheat, higher levels of iron, magnesium, and manganese may be associated with higher levels of bioavailable cadmium (30). Conflicts can also arise around processing and preparation techniques. Polishing rice is an effective technique for removing arsenic, but converting brown rice to white also reduces the iron and zinc content of the grain (31), decreases protein and fiber content, and may negatively alter the grain’s glycemic effect on the body (32). The US FDA suggests that most consumers manage the tension between nutrition and risk from arsenic in food by maintaining a diverse, balanced diet (33). Though this is sensible and familiar advice, it lacks specificity, and economic realities may make following diverse and balanced dietary patterns difficult or impossible for many consumers.

Health equity concerns around nutrition could be amplified when we add toxic metals to the equation. A participatory research project in Santa Ana, California demonstrated that even before considering dietary intake, the burden of environmental exposure to arsenic, cadmium, and lead may fall disproportionately on lower-income and Latino/a/e or Hispanic consumers (34). Disparities can also start to arise at the foundational level of drinking water. Many readers will recall the drinking water crisis that began in 2014 in the socioeconomically vulnerable, predominantly Black city of Flint, Michigan and likely compounded inequities in childhood lead exposure (35). Furthermore, consumers with less socioeconomic privilege may have limited choice and access to a wide variety of nutritious foods due to financial constraints. Urban gardening might help ameliorate this problem in some contexts, and consumers see a wide variety of benefits to these systems, including control over the addition of potentially toxic compounds like pesticides (36, 37). Unfortunately, urban garden soils can be contaminated with toxic metals (38, 39) and these metals can be taken up into crops (40). Consumers may suspect risks from soil contamination at the garden planning phase, and their concerns must be taken seriously (41). Without location-specific testing, better information about toxic metals in the full diet, and tools to interpret this data, this promising public health strategy for improving access to fresh produce in urban areas may be compromised.

## 3 Conclusions and proposed solutions

To begin addressing the challenges described above and enhance consumers’ protection from the effects of lower-dose chronic exposure to toxic elements, it is imperative to make data on toxic metals in foods more robust and accessible to provide actionable dietary guidance. Nutrition professionals require appropriate data and tools to help consumers determine which foods in which amounts are nutritious and safe to consume, balancing potentially competing dietary priorities as well as economic considerations. Although the scientific literature contains many reports on toxic metals in foods, the most readily available centralized and comprehensive survey of such information in the United States seems to be the FDA TDS. Creating a more interactive, user-friendly web interface for this database could be a good starting point. Such a database should also integrate what information is available on acceptable metal levels in foods and provisional tolerable intakes, though clearer guidance in these areas is needed. Although some links between chronic diseases and toxic elements are well-established (e.g., inorganic arsenic and cancer (4, 7); cadmium and kidney disease (3)), additional work should be done to clarify and communicate potential interactions or additive effects of low-level dietary toxic elements across the lifespan. Ideally, this data would ultimately be integrated with existing food composition databases, such as USDA’s FoodData Central, alongside nutrient information that is regularly accessed by nutrition professionals. However, such an effort might be met with reservations from companies in the food production space, which may be concerned about impacts on consumer perception and behavior. The health-focused rationale for expanding collection and use of data on toxic elements in food should be clearly communicated to corporations, and strategies to engage their support should be evaluated collaboratively. Although findings of elevated toxic element content in a given food could lead to negative press, perhaps rigorous practices within a company to monitor and mitigate toxic element content could win favor with consumers. Guidance from nutrition professionals about the continuum of risk from toxic elements and balancing the contributions of healthful food components may also help consumers make decisions based on a more nuanced assessment, rather than reacting solely out of fear to an increased awareness of dietary toxic elements.

As databases expand, it will be important to characterize variation in toxic metal content related to geographic origin, cultivation practices, and storage and processing conditions. A recent report on arsenic in rice suggests that this may be especially salient as climate change progresses and creates conditions that may impact uptake of toxic metals into foods (42). As illustrated by the discussion of urban garden soils, it will be particularly important for this type of work to be designed and assessed through a health equity lens. This includes consideration of the economic implications of characterizing particular foods or food sources as containing toxic elements – both for consumers who may not be able to afford alternatives, and to producers for whom sale of these foods is a sole or primary source of income. The prospect of adding spatial and social layers (and, ideally, their interactions) to the already-immense task of characterizing dietary chemical diversity naturally raises questions about analytical resources and data interpretation. There is no way around the need for real-world data. However, construction of a sufficiently large and robust dataset may eventually allow for development of tools (e.g., based on machine learning) that can “flag” foods of concern more rapidly. The time and materials needed for comprehensive analysis could be focused on foods where they are most likely to have the greatest impact within a given region or community.

**Figure 2:**
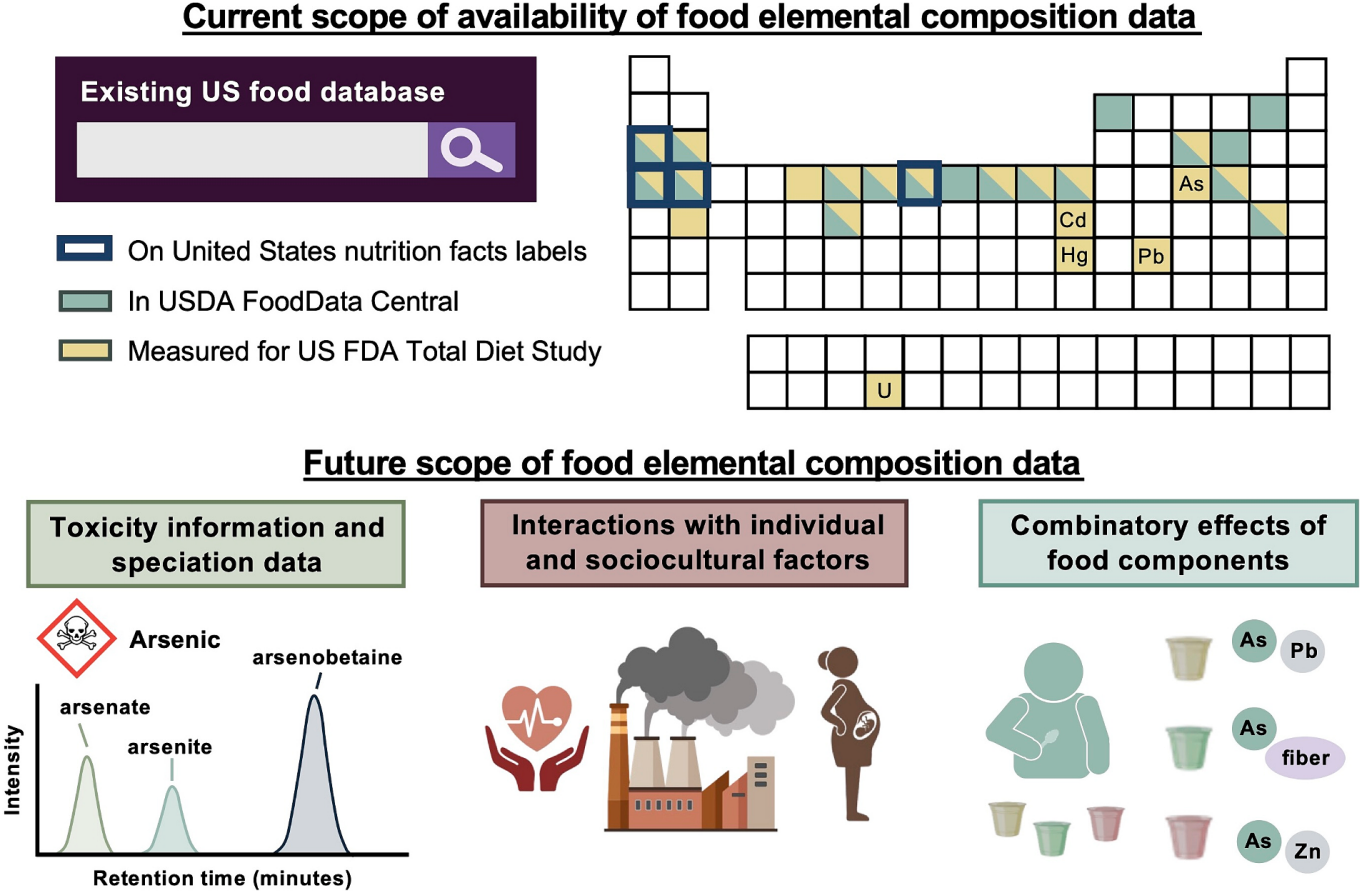
A comparison of the current and future scopes of food elemental composition data readily accessible to United States nutrition professionals. Many toxic elements can be measured, but still need to be incorporated into interactive food composition databases that are widely used by US nutrition professionals. To make this information actionable, toxicity thresholds and speciation data, or information about the chemical forms of an element present in food, are required for a number of toxic elements. Further work is also needed to differentiate guidance according to consumers’ environments and health statuses, and to understand interactions among food components for appropriately balanced recommendations. (USDA – United States Department of Agriculture; US FDA – United States Food and Drug Administration; Created in BioRender. Prenni, J. (2024) BioRender.com).

In the longer term, information about the distribution of chemical forms of metals – i.e., speciation data – in foods should also be incorporated into food composition databases. Some chemical forms of toxic metals, such as arsenic (43), are more dangerous than others; similarly, bioavailability of nutritive elements, such as zinc (44), can vary among chemical forms. Nutrition professionals already have a grounding in this concept – for example, the heme iron in meat is more bioavailable than the nonheme iron prevalent in plant foods (45). In the world of toxic metals, an analogous division exists between inorganic arsenic, which is classified as a known human carcinogen in the US EPA’s Integrated Risk Information System (7), and organic arsenic, which is not (note that the small organic species monomethylarsonic acid and dimethylarsinic acid are listed as possible human carcinogens by the WHO International Agency for Research on Cancer (46)). Speciation data is currently very limited in major US food composition databases (in the FDA TDS, arsenic speciation is performed on a small subset of foods (20)). These considerations are summarized in Figure 2.

Expanding the knowledge base of toxic elements in food will take time and a coordinated global effort, as will understanding how to appropriately integrate the data into nutrition advice. To the best of our understanding, the history of national dietary toxic metal regulation for US consumers is relatively recent: The EPA set enforceable limits for various toxic metals in drinking water over the course of the early 1990s (47, 48), and revised the allowable arsenic level to a lower value in 2001 (49). From a chemical analysis standpoint, food is much more complex than water, and the interface of food science and food policy is further complicated by the myriad sociocultural dimensions of food. The US FDA only finalized its (non-binding) infant rice cereal inorganic arsenic action level guidance of 100 ppb in 2020 (50). However, this recent movement should be taken as an encouraging sign that researchers’ continual improvement of techniques for food composition analysis – in tandem with epidemiological, toxicological, and nutrition-focused work – will allow further action in this space. Pressing concerns about escalating climate stress on global agricultural systems necessitates further advancement and broader application of these methods. The effort to better characterize and communicate about toxic metals in food is a critical investment in the promotion of a safer and more equitable understanding of nutrition.

## Data Availability

Publicly available US FDA TDS elements data is discussed, but not analyzed in detail. This data is available at: https://www.fda.gov/food/fda-total-diet-study-tds/fda-total-diet-study-tds-results.
